# Acoustic Radiation or Cavitation Forces From Therapeutic Ultrasound Generate Prostaglandins and Increase Mesenchymal Stromal Cell Homing to Murine Muscle

**DOI:** 10.3389/fbioe.2020.00870

**Published:** 2020-07-28

**Authors:** Rebecca M. Lorsung, Robert B. Rosenblatt, Gadi Cohen, Joseph A. Frank, Scott R. Burks

**Affiliations:** ^1^Frank Laboratory, Department of Radiology and Imaging Sciences, NIH Clinical Center, Bethesda, MD, United States; ^2^Intramural Research Program, National Institute of Biomedical Imaging and Bioengineering, Bethesda, MD, United States

**Keywords:** pulsed focused ultrasound, low intensity pulsed ultrasound, prostaglandin, cavitation, acoustic radiation force, mesenchymal stromal cell, regenerative medicine, cell therapy

## Abstract

Non-ablative ultrasound (US)-based techniques to improve targeted tropism of systemically infused cell therapies, particularly mesenchymal stromal cell (MSC), have gained attention in recent years. Mechanotransduction following targeted US sonications have been shown to modulate tissue microenvironments by upregulating cytokines, chemokines, and trophic factors in addition to vascular cell adhesion molecules (CAM) that are necessary to promote tropism of MSC. While numerous US treatment parameters have demonstrated increased MSC homing, it remains unclear how the different mechanical US forces [i.e., acoustic radiation forces (ARF) or cavitation forces] influence tissue microenvironments. This study sonicated murine muscle tissue with pulsed focused ultrasound (pFUS) at 0.5 or 1.15 MHz each over a range of US intensities. Following sonication, tissue was assayed for the prostaglandins (PG) PGH_2_ and PGE_2_ as indicators of microenvironmental changes that would support MSC tropism. PGH_2_ and PGE_2_ levels were correlated to physical pFUS parameters and acoustic emissions measured by hydrophone. While ARF (pFUS with absence of cavitation signatures) was sufficient to increase PGH_2_ and PGE_2_, non-linear curve fitting revealed a frequency-independent relationship between prostaglandin production and mechanical index (MI), which accounts for increased cavitation probabilities of lower frequencies. The prostaglandin data suggested molecular changes in muscle would be particularly sensitive to cavitation. Therefore, low-intensity pulsed ultrasound (LIPUS) at 1 MHz was administered with low ARF (MI = 0.2) in combination with intravenous (IV) infusions of microbubble (MB) contrast agents. This combination upregulated prostaglandins and CAM without ultrasound-mediated microbubble destruction and ultimately promoted tropism of IV-infused MSC. This study revealed that accentuating non-destructive MB cavitation by US using parameters similar to diagnostic US contrast imaging increased MSC homing. Such approaches are particularly attractive to overcome clinical translation barriers of many still-experimental US parameters used in previous stem cell tropism studies.

## Introduction

Non-ablative therapeutic ultrasound (US), including pulsed focused ultrasound (pFUS) or unfocused low-intensity pulsed ultrasound (LIPUS), is of increasing interest as a biomedical tool to alter tissue microenvironments in regenerative medicine. An area of particular interest is the potential for US-based approaches to improve stem cell therapies, and mesenchymal stromal cell (MSC) therapies in particular. Therapeutic MSC that are systemically infused are often required to then “home” to pathological tissues and therapeutic efficacy has historically been hindered by inefficient homing and accumulation of MSC to pathological sites (De Becker and Riet, 2016). Non-ablative US sonications induce structural and molecular changes in targeted tissues that can increase homing of systemically-infused (MSC) therapeutics to sonicated tissues [for review see Liu et al. (2020)]. US-induced MSC homing has demonstrated therapeutic potential in experimental animal models of critical limb ischemia (Tebebi et al., 2017), myocardial infarction (Sun et al., 2020), acute liver injury (Sun et al., 2018), diabetic nephropathy (Zhang et al., 2013), and acute kidney injury (Burks et al., 2015, 2018). Evidence suggests MSC tropism mechanisms are analogous to those of leukocyte homing which rely on endothelial cell activation and local parenchymal gradients of molecular chemoattractants (Karp and Leng Teo, 2009; Ullah et al., 2019). Previous US studies characterized the molecular microenvironmental changes in tissues post-sonication and overwhelmingly observe increased expression of cytokines, chemokines, trophic factors and adhesion molecules (CAM) which would facilitate stem cell tethering and rolling across the activated endothelium followed by diapedesis into the parenchyma (Liu et al., 2020). Several of our previous studies have investigated microenvironmental changes alone (Burks et al., 2011) or in context of stem cell homing to skeletal muscle and kidney using 1 MHz pFUS at a peak negative pressure (PNP) of ∼4 MPa without microbubble (MB) infusions (Ziadloo et al., 2012; Burks et al., 2013, 2017; Tebebi et al., 2015). We have demonstrated that microenvironmental changes to facilitate stem cell homing require nuclear factor kappa B (NFκB) activation and subsequent cyclooxygenase-2 (COX2) upregulation (Tebebi et al., 2015; Burks et al., 2017).

Specific microenvironmental changes resulting from mechanotransduction of US have received substantial attention, yet it remains unclear how the mechanical effects of US interact with tissues elicit these molecular responses. We previously demonstrated in skeletal muscle and kidney that pFUS at the above parameters mechanically activates the transient receptor potential channel 1 (TRPC1), which subsequently activates voltage gated calcium channels to initiate a calcium-dependent increase in COX2 expression (Burks et al., 2019). At those pFUS parameters, formation and cavitation of bubbles from dissolved tissue gases was not detected and viscoelastic modeling suggested that tissue deformations generated by acoustic radiation forces (ARF) were sufficient to increase COX2. However, wide range of US treatment modalities and parameters such as the use of pFUS or LIPUS, different US frequencies and intensities, and the use of intravenous (IV) MB to enhance cavitation forces all have been shown to generate microenvironmental changes to induce targeted MSC tropism (Liu et al., 2020). Numerous stem cell homing studies have employed extreme cavitation effects by ultrasound-mediated microbubble destruction (UMMD) to modulate tissue microenvironments (Ling et al., 2013) and achieve MSC homing. However, UMMD induces tissue damage and therefore would not be ideal for regenerative medicine applications. Limited understanding of the mechanical US forces that generate the desired bioeffects for MSC homing has prevented rational optimization of US techniques to facilitate their clinical translation and ability to improve cellular therapies.

The aim of this study was to investigate different US parameters and their bioeffects relevant to MSC homing in skeletal muscle. This study examined molecular changes in murine skeletal muscle following sonication at different frequencies and intensities, with and without IV MB infusions, to emphasize different mechanical US forces while measuring acoustic emissions. Having previously identified COX2 expression as a critical molecular mediator of US bioeffects (Burks et al., 2015, 2017, 2019), we directly investigated COX2 activity following sonication by quantifying prostaglandin-H_2_ (PGH_2_), the direct metabolite of COX2, and PGE_2_, which our previous studies identified as a potential effector molecule and is known to enhance MSC homing (Lu et al., 2017). Investigating US-induced prostaglandin production as a function of frequency and intensity revealed a strong correlation between prostaglandin levels and mechanical index (MI), suggesting cavitation forces are particularly effective at eliciting bioeffects in tissues. While cavitation did appear explicitly required to induce molecular changes in tissues (Burks et al., 2019), we investigated whether LIPUS sonications that remain below the FDA-approved MI limit of 1.9 and utilized mild cavitation effects (i.e., not UMMD) with approved US MB contrast agents (e.g., Definity^®^ or Optison^TM^) would be effective to induce MSC homing to skeletal muscle. If such approaches could be successfully developed, they would resemble parameters similar to diagnostic contrast imaging and may face fewer regulatory hurdles toward clinical translation.

## Materials and Methods

### Animals

All animal studies were approved by the Animal Care and Use Committee of the Clinical Center and procedures were performed according to National Research Council’s Guide for the Care and Use of Laboratory (2011). Female NIH Swiss mice (8–12 weeks old; Charles River Laboratories, Wilmington, MA, United States) were housed with free access to food and water. Hair was removed with depilatory cream 24 h before sonication. Mice were anesthetized with 2–3% isofluorane in O_2_ for all ultrasound treatments, except experiments using MB, which used room air as the carrier gas.

### Cell Culture and Infusions

Human MSC were cultured in α-minimum essential medium supplemented with 20% fetal bovine serum and 1% penicillin/streptomycin solution (ThermoFisher, Waltham, MA, United States). Cell-surface marker expression was previously characterized (Ziadloo et al., 2012). For infusions into mice, MSC (Passage 6) were trypsinized and resuspended at a concentration of 10^7^ cells/mL in Hank’s balanced salt solution (HBSS) containing 10 U/mL sodium heparin.

### Ultrasound Treatments

#### Pulsed Focused Ultrasound

Pulsed Focused Ultrasound for molecular and histological analyses was administered with a VIFU 2000 (Alpinion Medical Systems, Bothell, WA, United States) under ultrasound imaging guidance in degassed water at 37°C. Hamstrings were treated with either a 0.5 or 1.15 MHz transducer. Ultrasound at 0.5 MHz was administered using the following parameters: peak negative pressures (PNP) of 1, 2, 3.5, or 5 MPa, 5 Hz pulse repetition frequency (PRF); US burst length 10 ms, 5% duty cycle (d.c.); 100 pulses per treatment point. The entire hamstring was treated using elemental spacing of 2.5 mm. Ultrasound at 1.15 MHz was administered using PNP of 2, 4, 6, or 8 MPa and elemental spacing of 2 mm. The distances between treatment points were determined such that the no two spots overlapped within the -6 dB cutoff of the focal diameter. Three-dimensional intensity mapping of each transducer was provided by the manufacturer and performed in a tank of degassed water.

#### Low Intensity Pulsed Ultrasound

Low Intensity Pulsed Ultrasound was administered with a 2.86-cm linear transducer (Olympus, Shinjuku, Tokyo, Japan) operating at 1 MHz using the following parameters: peak rarefaction amplitude of 200 kPa (MI = 0.2); pulse repetition frequency 1 Hz; US burst 50 ms, 5% duty cycle; 200 pulses. LIPUS instrumentation was controlled using LabView (National Instruments, Austin, TX, United States). Catheters were inserted into the lateral tail veins and Definity^®^ (Lantheus Medical Imaging, Billerica, MA, United States) perflutrin lipid microspheres were infused at a concentration of 10 μL/kg body weight immediately before beginning sonication. Approximately 1 h after sonication, mice were given an IV injection of sodium nitroprusside (1 mg/kg) followed immediately by IV infusion of 5 × 10^5^ MSC.

### Passive Cavitation Detection

Acoustic spectra were acquired *in vivo* using an LP100 magnetic-resonance (MR)-guided FUS system (FUS Instruments, Toronto, ON, United States). Transducers for the LP100 contained in-line lead-titonate-zirconate hydrophones tuned to ∼700 kHz. Treatment planning was guided using standard T_2_-weighted magnetic resonance imaging sequences using an Acheiva 3T scanner (Phillips, Amsterdam, Netherlands). Sonications were performed as single treatment spots in the hamstrings at operating frequencies of 548 kHz or 1.12 MHz which were correlated to molecular and histological data acquired using pFUS at 0.5 kHz or 1.15 MHz, and LIPUS data at 1 MHz. Other sonication parameters (PNP, pulse length, dc, etc.) at 548 kHz were identical to the 500 kHz pFUS treatments above, while parameters for the 1.12 MHz treatments were identical to the 1.15 MHz pFUS treatments or 1.0 MHz LIPUS treatments with microbubbles outlined above.

### Proteomic Analyses

Hamstrings for molecular analyses were harvested (*n* = 5–6/treatment group) and flash frozen in liquid N_2_. Tissue was homogenized in ice cold Tris-buffered saline (TBS) containing 0.05% Tween-20, protease inhibitor cocktail (Roche, Mannheim, Germany), and 20 μg/mL indomethacin. Insoluble material was removed by centrifugation at 15,000 rpm for 20 min at 4°C. Total protein content of each sample was determined using a bicinchoninic acid assay (ThermoFisher). Prostaglandins H_2_ and E_2_ were measured using enzyme-linked immunosorbent assays (ELISA) against Prostaglandin E_2_ (R&D Systems, Bio-Techne, Minneapolis, MN, United States) or prostaglandin H_2_ (antibodies-online.com, Limerick, PA, United States).

### Immunohistochemistry

Hamstrings for immunohistochemistry (IHC) (*n* = 3/treatment group) were harvested following cardiac perfusion with 10% neutral buffered formalin. Tissue was fixed overnight at 4°C, then dehydrated in 30% sucrose. Hamstrings were embedded in optimum cutting temperature (OCT) compound and sectioned at 10 μm thickness. Immediately before staining, frozen sections were baked at 60^*o*^C for 15 min then rinsed with PBS. Antigen retrieval was performed by incubating slides with Proteinase K at 37°C for 20 min, blocked using Superblock (10 min), then incubated with primary antibodies for 1 h. Primary antibody against human mitochondria (#ab92824) was from Abcam (Cambridge, MA, United States) and antibodies against CD54 (#116101), CD106 (#105701), CD62l (#104402), and CD62p (#148301) were from Biolegend (San Diego, CA, United States). A 1-h incubation was performed with species-specific secondary antibodies. Samples were mounted in ProLong Gold Antifade mounting medium (Thermo Fisher Scientific, Wilmington, MA, United States). Fluorescence was visualized with an Axio Observer (Carl Zeiss AG, Oberkochen Germany) or Aperio ScanScope FL (Leica Biosystems, Wetzlar, Germany) with appropriate excitation and emission filters.

### Data Processing

All data are presented as mean ± standard deviation with Prism 8 (GraphPad Software, Inc., La Jolla, CA, United States). Potential outliers were tested for using the ROUT method, *Q* = 0.05%. One-way analysis of variance (ANOVA) with Dunnett’s *post hoc* analyses were used to for statistical comparisons. Ultrasound parameters of intensity and PNP were provided by transducer manufacturers. ARF was calculated using equation (1) and MI was calculated using equation (2). Prostaglandin response curves were fitted using quadratic equations and curves compared using the extra sum-of-squares best fit *F* tests. Hydrophone data were processed offline in MATLAB (Mathworks, Natick, MA, United States). Electrical signals were transformed using Fast Fourier Transforms (FFT). To quantify harmonic emissions, FFT amplitude values within 20 kHz windows around harmonics (i.e., harmonic frequency ± 10 kHz) were integrated. Integrated values from 20 kHz windows around 2f, 3f, 4f, and 5f were totaled. The half-harmonic emission was represented as the integral of a 20 kHz window around the half-harmonic value. Broadband emissions had 100 kHz windows around the half-harmonic, fundamental frequency, and harmonics removed. Broadband emissions were then represented as the integral of inter-harmonic noise. MSC were quantified following IHC for human mitochondria. MSC were manually counted in a total of 90 high-power fields-of-view (FOV) [10 FOV per section, from three sections per hamstring and three hamstrings (i.e., mice) per treatment group].

(1) $$\text{ARF}=\frac{2\alpha I_{\text{SATP}}}{c}$$

*α* is the US absorption coefficient of muscle (0.149 Np/cm) (Nassiri et al., 1979).

*I*_*SATP*_ is the US spatial-average temporal-peak intensity (W/cm^2^).

*c* is the speed of sound (1,540 m/s).

(2) $$\mathrm{MI}=\frac{\text{PNP}}{\sqrt{F_{0}}}$$

PNP is the peak negative pressure (MPa).

*F*_0_ is the fundamental ultrasound frequency (MHz).

## Results

### pFUS Increases PGH_2_ and PGE_2_ for 24 h Following pFUS Treatment

**FIGURE 1:**
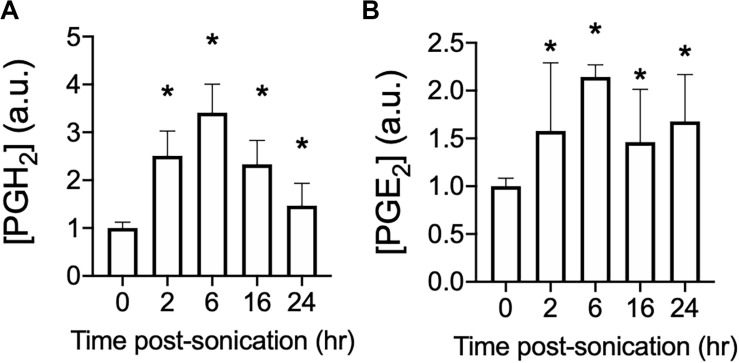
Time course of PGH_2_ and PGE_2_ following pFUS to skeletal muscle. Mouse hamstrings (*n* = 6 per time point) were treated with pFUS at 1.15 MHz and 8 MPa. Hamstrings were harvested at various time points and relative changes in PGH_2_ **(A)** and PGE_2_ **(B)** are shown. Asterisks represent statistically significant differences (*p* < 0.05 by ANOVA) compared to sham pFUS treatment (0 h time point).

pFUS at 1.15 MHz and 8 MPa PNP was administered biilaterally to mouse hamstrings [*N* = 3 mice (6 hamstrings) per time point] and PGH_2_ (Figure 1A) and PGE_2_ (Figure 1B) production were measured at various times following sonication. This PNP was chosen as a maximal intensity experiments to reliably increase prostaglandin production and identify if production levels peak at a specific time following pFUS. For both prostaglandins, significant elevations (*p* > 0.05 by ANOVA) were detected at 2, 6, 16, and 24 h post-sonication. Peak levels of both PGH_2_ and PGE_2_ were detected at 6 h after treatment and therefore, 6 h was chosen as the euthanasia timepoint for all subsequent prostaglandin measurements.

### Prostaglandin Increases Are Directly Related Sonication Peak Negative Pressure at 0.5 or 1.15 MHz

**FIGURE 2:**
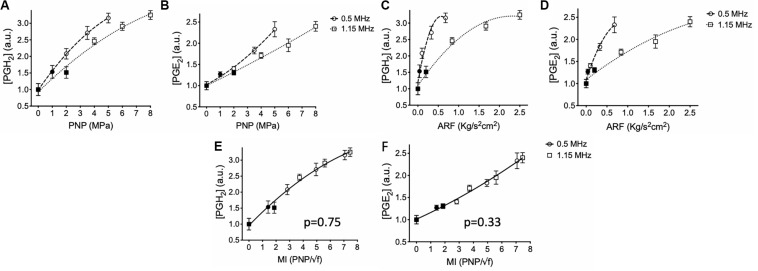
Prostaglandin production in skeletal muscle following sonication at different intensities and frequencies. Mouse hamstrings (*n* = 6 per treatment group) were sonicated at 0.5 or 1.15 MHz and relative expression of PGH_2_ **(A,C,E)** and PGE_2_ **(B,D,F)** at 6 h post-sonication are shown. Changes in prostaglandins for each frequency were then plotted as functions PNP **(A,B)**, ARF **(C,D)**, and MI **(E,F)**. Significantly elevated prostaglandin levels (*p* < 0.05 by ANOVA) compared to sham sonication levels are indicated by open symbols while filled symbols indicate statistically similar values. Each curve was fit using quadratic equations and statistical comparisons between curves were performed by extra sum of squares F tests. Curves compared between frequencies in panels **(E)** and **(F)** were found to be significantly similar (*p* values shown) and thus data are presented with single curve fits for data sets from both frequencies.

pFUS was administered unilaterally to murine hamstrings at either 1.15 or 0.5 MHz [*N* = 6 mice (6 hamstrings) per treatment group]. Hamstrings were treated with 0.5 MHz at 1, 2, 3.5, or 5 MPa PNP, while mice treated with 1.15 MHz received PNP of 2, 4, 6, or 8 MPa. A sham control group of mice received 0 MPa treatments (no power to the transducer). Levels of PGH_2_ and PGE_2_ increased with greater PNP at both frequencies (Figures 2A,B). At 1.15 MHz, PNP = 2 MPa sonication did not significantly elevate PGH_2_ or PGE_2_ compared to sham treatment, however, sonicating at 4, 6, or 8 MPa significantly increased prostaglandins in the muscle (*p* < 0.05 by ANOVA). Sonication at 0.5 MHz significantly elevated levels of both PGH_2_ and PGE_2_ at 2, 3.5, and 5 MPa (*p* < 0.05 by ANOVA), but not at PNP = 1 MPa. PNP-dependent response curves for PGH_2_ and PGE_2_ at each frequency were best fit using quadratic equations and statistical comparison of fits for response curves for each prostaglandin were found to be significantly different between the two frequencies (*p* < 0.0001 for PGH_2_ and PGE_2_ by extra sum-of-squares *F* tests).

Statistical curve comparisons suggesting PGH_2_ and PGE_2_ production are not strictly a function of PNP, therefore prostaglandin quantities were also plotted and fitted as a function of ARF (Figures 2C,D). Similarly, molecular response curves differed significantly for each frequency (*p* < 0.0001 for PGH_2_ and PGE_2_ by extra sum-of-squares F tests). Sonication at 0.5 MHz was able to produce greater quantities of PGH_2_ and PGE_2_ at lower ARF than sonications at 1.15 MHz. For example, 5 MPa sonications at 0.5 MHz produced similar levels of prostaglandins as 8 MPa at 1.15 MHz despite generating only ∼25% the ARF. These data suggested that molecular pFUS responses did not correlate entirely with ARF. Therefore, PGH_2_ and PGE_2_ values were plotted and fitted against MI (Figures 2E,F). Statistical comparison of response curves revealed that response curves for PGH_2_ and PGE_2_ did not significantly differ as functions of sonication frequency (*p* = 0.75 for PGH_2_; *p* = 0.33 for PGE_2_ by extra sum-of-squares *F* tests) and that PGH_2_ and PGE_2_ datasets could be fit by single, frequency-independent response curves.

**FIGURE 3:**
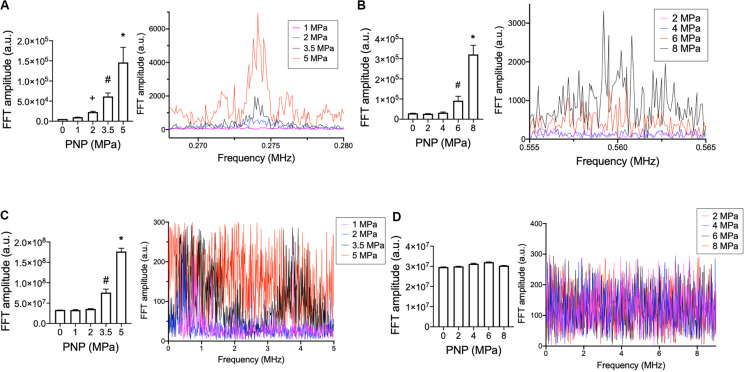
Acoustic emissions from hamstring sonications at 0.548 or 1.125 MHz at different PNP. Statistical summaries and representative spectra are shown measuring half-harmonic **(A,B)** and broadband **(C,D)** emissions at different PNP for 0.548 MHz **(A,C)** and 1.125 MHz **(B,D)**. Graphs represent average values from 100 sonication pulses. Symbols indicate statistical differences (*p* < 0.05) from all other values when compared by ANOVA.

Lastly, acoustic emissions were measured for sonications at 0.548 and 1.125 MHz to contextualize molecular data acquired at 0.5 and 1.15 MHz, respectively. Increased half-harmonic emissions at 1.125 MHz were not observed at 2 or 4 MPa but were observed at 6 and 8 MPa (Figure 3A). Increased broadband emissions at 1.125 MHz were not detected at any PNP (Figure 3C). For 0.548 MHz, increases in half-harmonic emissions were observed 2, 3.5, and 5 MPa, but not 1 MPa (Figure 3B). Increased broadband emissions were detected only at 3.5 and 5 MPa (Figure 3D).

### LIPUS With MB Infusions Alters the Muscle Microenvironment and Increases MSC Homing

**FIGURE 4:**
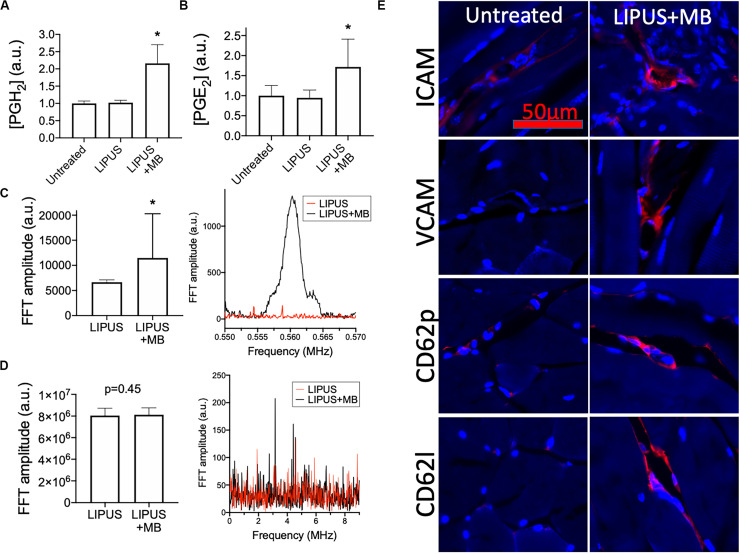
LIPUS + MB alters skeletal muscle microenvironment and increases homing of systemically infused MSC. Mice (*n* = 6 per group) were given LIPUS + MB or LIPUS alone and relative expression of PGH_2_ **(A)** and PGE_2_ **(B)** were quantified at 6 h post-sonication. Only LIPUS + MB significantly increased prostaglandin levels (*p* < 0.05 by ANOVA). LIPUS + MB increased half-harmonic emissions **(C)** (*p* < 0.05 by *t*-test) without increasing broadband emissions **(D)** (*p* = 0.45 by *t*-test). Immunohistochemistry for CAM **(E)** reveal upregulation of ICAM, VCAM, CD62p, and CD62l (all shown in red) at 6 h-post sonication compared to untreated muscle. Asterisks indicate statistical significance (*p* < 0.05) by ANOVA in **(A,B)** or by *t*-test in **(D)**.

**FIGURE 5:**
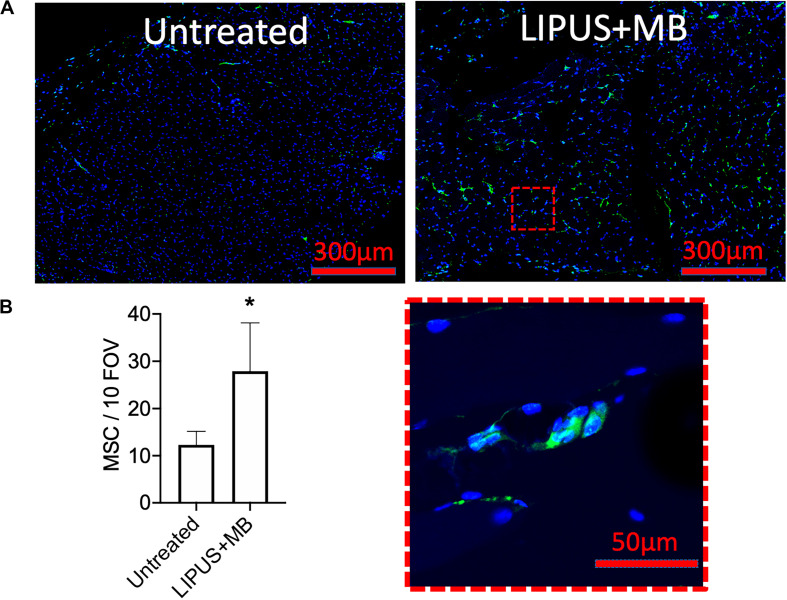
LIPUS + MB increases homing of systemically infused MSC. Mice (*n* = 3 per group) received 5 × 10^5^ human MSC IV following LIPUS + MB to one hamstring. **(A)** IHC for human mitochondria revealed increased presence of MSC (green); inset shows high magnification view of MSC) in hamstrings receiving LIPUS + MB compared untreated contralateral hamstring tissue. Quantification in panel **(B)** counted MSC from 10 high-powered fields-of-view from 3 tissue section per animal (asterisk indicates *p* < 0.05 by *t*-test).

LIPUS sonications (1 MHz, 0.2 MPa) coupled with MB infusions (10 μL/kg of Definity^®^ IV) were administered unilaterally to murine leg muscle (*N* = 6 mice). Compared to LIPUS-sonicated control muscle tissue without MB, LIPUS + MB significantly increased levels of PGH_2_ and PGE_2_ (*p* < 0.05 by ANOVA) (Figures 4A,B). Alternatively, muscle treated with LIPUS alone (no MB infusions) did not exhibit elevated PGH_2_ or PGE_2_ production compared to controls (*p* > 0.05 by ANOVA). Acoustic spectra at 1.12 MHz revealed increased half-harmonic emissions during sonications with MB compared to sonication alone (Figure 4C). Moreover, increased broadband emissions were not detected when MB infused (Figure 4D). Immunohistochemistry revealed immunoreactivity for several CAM. ICAM, VCAM, CD62p (p-selectin), and CD62l (l-selectin) were upregulated in muscle tissue treated with LIPUS + MB (Figure 4E) compared to controls. Having observed increased prostaglandin production and increased levels of CAM with LIPUS + MB, another cohort of mice was treated and MSC (10^5^ per mouse) were injected IV immediately after LIPUS + MB treatments (*N* = 3 mice per group). Mice were euthanized 24 h post-treatment and human mitochondrial immunostaining revealed significantly greater quantities of MSC in sonicated legs (∼2.5-fold more) compared to untreated contralateral muscle (Figure 5).

## Discussion

The major findings of this study are that pFUS-induced microenvironmental changes in skeletal muscle occur following sonications that generate sufficient ARF *or* cavitation forces. However, the magnitude of molecular responses are not primarily influenced by ARF. Tissue microenvironmental changes and increased MSC homing were observed using unfocused LIPUS with MB infusions that emphasize cavitation forces. The LIPUS + MB represents an attractive option for image-guided SC homing to skeletal muscle given that is both amenable to regenerative medicine approaches (i.e., not UMMD) and within a framework of FDA-approved sonication techniques (e.g., MI ≤ 1.9).

Non-thermal US-based approaches alter tissue microenvironments and increase MSC homing have been demonstrated using numerous ultrasound parameters (Liu et al., 2020), but without clear optimization for regenerative medicine approaches. The lack of understanding how US interacts with tissues to produce the desired bioeffects has precluded rational optimization and potential clinical translation of techniques.

Our group has studied microenvironmental changes (Burks et al., 2011) and MSC homing to tissues extensively but using only a limited set of US parameters. Primarily, we have used non-thermal pFUS usually at PNP of 4 MPa (MI = ∼4) without infusions of IV MB. These parameters were based on previous work demonstrating increased vascular permeability (Hancock et al., 2009) and that strongly suggested the occurrence of molecular biological changes necessary to enhance permeability and retention of MSC into tissues. Those pFUS parameters have been sufficient to induce bioeffects in muscle and kidney to generate microenvironmental changes and upregulate CAM primarily through mechanotransduction of ARF without the presence of endogenous cavitation (Burks et al., 2019). However, this approach has limited translational potential as non-ablative pFUS remains unapproved and utilized MI above current FDA limits for diagnostic US.

The aim of this study was to investigate different US parameters and characterize their relationships with microenvironmental changes in skeletal muscle. Muscles were sonicated over ranges of intensities using two different US frequencies. This allowed varied US parameters to emphasize either ARF or cavitation forces and could potentially provide insight into how US bioeffects are generated. For this study, production of the prostaglandins PGH_2_ and PGE_2_ were investigated serving as proxy indicators of microenvironmental changes. We have previously demonstrated that COX2 upregulation by NFκB is a critical component of the molecular signaling cascade within 4–6 h following pFUS at 4 MPa (Tebebi et al., 2015; Burks et al., 2017, 2019). While we have investigated COX2 expression in multiple studies, we have never investigated enzyme activity by quantifying metabolites. Here, we examined levels of PGH_2_ which is the direct metabolite of COX2 and serves as the precursor to numerous other prostaglandins including PGE_2_. Quantifying PGE_2_ is of particular interest because it has been implicated by our previous *in silico* analyses as an important effector molecule to generate the US bioeffects to increase MSC homing (Tebebi et al., 2015). Indeed, PGE_2_ alone has been previously demonstrated to increase local homing of MSC (Lu et al., 2017).

Production of both PGH_2_ and PGE_2_ in sonicated skeletal muscle was related to PNP at 0.5 or 1.15 MHz, but specific relationships for both prostaglandins concentrations differed with frequency. Physical US forces of ARF and cavitation are dependent on frequency (Izadifar et al., 2017). PNP and ARF require greater intensities to achieve at 0.5 MHz, while cavitation probability increases at 0.5 MHz. Analyzing prostaglandin production as a function of ARF rather than PNP was particularly insightful. ARF alone was previously demonstrated as sufficient to generate bioeffects that increase MSC homing (Burks et al., 2019) and confirmed by the prostaglandin data in this study. Significant prostaglandin production at 1.15 MHz was observed at PNP ≥ 4 MPa. At 4 MPa specifically, 1.15 MHz US does not generate detectable cavitation signatures and further demonstrates ARF alone can induce prostaglandin production. However, comparing ARF generated at each frequency, no PNP at 0.5 MHz generated as much ARF as 4 MPa at 1.15 MHz, yet some PNP at 0.5 MHz were capable of producing prostaglandin levels in excess of what was observed using 4 MPa at 1.15 MHz. In light of the observation that 0.5 MHz generates prostaglandins with little ARF and that cavitation probability is inversely related to US frequency, we sought a relationship between prostaglandins and US parameters that account for cavitation. A frequency-independent relationship was observed between prostaglandin production and MI. MI is a relative measure of non-thermal US forces that accounts for increased cavitation probability at lower US frequencies (Szabo, 2014). This unified relationship between frequencies further suggested that endogenous cavitation generated by pFUS is an important physical force to induce relevant bioeffects. Indeed, cavitation from US (whether endogenous or from infused MB) generate significant bioeffects. Further correlations between prostaglandin production and acoustic spectral features revealed that cavitation signatures such as increased half-harmonic or broadband emissions were only detectable at PNP ≥ 6 MPa at 1.125 MHz with no detectable cavitation signature at 4 MPa. However, at 0.548 MHz, half-harmonic or broadband emissions were detectable at PNP ≥ 2 MPa. While not a conclusive demonstration, these data globally suggest that increased prostaglandin production at lower frequencies are driven primarily by cavitation forces and the possibility that muscle tissue is actually more sensitive to cavitation than ARF.

To explore the potential that acoustic cavitation specifically induces US-related bioeffects and MSC homing to muscle, we performed LIPUS in combination with IV MB infusions. MB serve as cavitation nuclei and generate forces at lower US intensities than those originating from endogenous bubble formation and cavitation. LIPUS at 1 MHz and 0.2 MPa did not generate microenvironmental changes alone, but did when MB were present in the circulation. LIPUS + MB was also found to upregulate endothelial CAM, including p-selectin. These histological and microenvironmental changes permit increased tropism and transmigration of MSC from the vasculature into the muscle.

Having investigated different US parameters to induce the microenvironmental changes necessary for MSC homing and determining that LIPUS + Definity^®^ MB increases MSC homing have important implications for clinical translation. To date, this study represents the only demonstration of MSC homing entirely using parameters and reagents in a manner that would be ideal for cellular therapy in regenerative medicine. For instance, non-ablative focused ultrasound currently remains unapproved even at low intensities in medical practice. Other demonstrations of MSC homing have relied on unapproved MB formulations or used US to generate UMMD. UMMD effectively induces MSC homing but can cause vascular damage and microhemorrhage which is not ideal for regenerative medicine.

The LIPUS + MB parameters used in this study are not dissimilar to those used in diagnostic US imaging (i.e., MI < 1.9 and an FDA-approved US contrast agent). US contrast imaging is frequently employed while ignoring potential bioeffects of MB cavitation, though studies are beginning to reveal molecular and cellular changes in tissues using standard contrast imaging protocols (Sen et al., 2015). For example, diagnostic US contrast imaging of the spleen was found to stimulate splenic microenvironmental changes that alter the neuroimmune axis and better prevent renal ischemic-reperfusion injuries (Gigliotti et al., 2013). Although 1 MHz US is lower than what is conventionally used for imaging and MB cavitation behaviors are among other things, a function of US frequency (Giesecke and Hynynen, 2003), further investigations into MSC homing using imaging transducers operating at more relevant frequencies is warranted.

In conclusion, US-based techniques to improve MSC homing are promising and being widely investigated as tools to overcome what is frequently inefficient homing of cellular therapeutics to diseased tissues. Tissues have been widely demonstrated to be sensitive to a variety of US-induced forces and respond with altered expression of chemoattractant and cell adhesion molecules that enhance homing permeability retention of infused MSC. While more sophisticated US techniques are being clinically investigated for cellular therapies (NCT02756884, clinicaltrials.gov), they still lack FDA approval. This study reveals that approved treatment parameters with demonstrated safety are sufficient to increase MSC homing and could be translated into clinical trials to enhance cellular therapy trials in regenerative medicine or other diseases.

## Data Availability Statement

The raw data supporting the conclusions of this article will be made available by the authors, without undue reservation.

## Ethics Statement

The animal study was reviewed and approved by NIH Clinical Center Animal Care and Use Committee.

## Author Contributions

RL, JF, and SB conceptualized research and designed experiments and wrote the manuscript. RL, RR, GC, and SB performed experiments. RL and SB analyzed data. All authors read and approved the submitted version of the manuscript.

## Conflict of Interest

The authors declare that the research was conducted in the absence of any commercial or financial relationships that could be construed as a potential conflict of interest.
